# Parkinsonics: A Randomized, Blinded, Cross-Over Trial of Group Singing for Motor and Nonmotor Symptoms in Idiopathic Parkinson Disease

**DOI:** 10.1155/2022/4233203

**Published:** 2022-09-20

**Authors:** Ankur Butala, Kevin Li, Aathman Swaminathan, Susan Dunlop, Yekaterina Salnikova, Bronte Ficek, Brandon Portnoff, Michael Harper, Bailey Vernon, Bela Turk, Zoltan Mari, Alexander Pantelyat

**Affiliations:** ^1^Department of Neurology, Johns Hopkins University School of Medicine, Baltimore MD 21218, USA; ^2^Krieger School of Arts and Sciences, Johns Hopkins University, Baltimore MD, USA; ^3^Moser Center for Leukodystrophies, Kennedy Krieger Institute, Johns Hopkins Medical Institutions, Baltimore MD, USA

## Abstract

**Introduction:**

Parkinson's disease (PD) frequently causes communication difficulties due to various voice impairments and there are few treatment options for vocal/communication complaints. We assessed the effects of weekly group singing on PD patients' objective vocal and motoric function, cognition, mood, self-efficacy, and quality of life.

**Methods:**

Thirty-two participants were randomly assigned to either a singing group or a facilitated discussion group weekly over 12 weeks. After 12 weeks, participants crossed over for an additional 12 weeks. Evaluations were performed at baseline and every six weeks for 30 weeks. Objective voice measures included volume/loudness (decibels), held vowel duration, jitter, shimmer, and harmonic-to-noise ratio. Additional outcome measures included patient-centered quality of life, voice-related quality of life, MDS-UPDRS, Montreal Cognitive Assessment, and questionnaires assessing depression, self-efficacy, and overall well-being.

**Results:**

Twenty-six participants (16 M/10F; Hoehn & Yahr stage 2.3 (range 2–3); and age 68.6 (55–89)) completed the study. Across participants in both groups (intention-to-treat analyses), there was significant improvement from baseline in average loudness on the Cookie Theft picture description at 24 weeks (end of interventions), corresponding with improved minimal reading volumes at 24 weeks and 30 weeks (end of study). Similarly, there were improvements in minimal loudness on Rainbow passage reading at 24 and 30 weeks. There were improvements observed in the Emotional Well-Being (mean delta −12.7 points, *p* = 0.037) and Body Discomfort (mean delta −18.6 points, *p* = 0.001) domains of the PDQ-39 from baseline to week 24 in the overall cohort and greater improvement in the Communication domain for Group S than Group D after 12 weeks of singing (delta −12.9 points, *p* = 0.016). Baseline differences between the participant groups (age, gender, Hoehn & Yahr stage, and several voice loudness measures) and observed improvements during the weekly discussion group period limited our ability to attribute all of the above results specifically to singing (per-protocol analyses). No significant changes in other assessed outcome measures were found.

**Conclusions:**

Weekly group singing may improve some aspects of conversational voice volume and quality of life in PD. Some improvements were sustained at least six weeks after interventions ended. Further investigations of the mechanism of benefit and randomized controlled studies (without crossover) to assess the longitudinal effects of singing in PD are necessary.

## 1. Introduction

Idiopathic Parkinson's disease (PD) is a chronic and progressive multisystem neurodegenerative disorder resulting in bradykinesia, rigidity, resting tremor, postural instability, and additional motor and nonmotor symptoms [1, 2]. Although different pharmacotherapies exist as symptomatic treatments, at present, there is no approved disease-modifying therapy. Physical therapy (PT) of various types has been demonstrated to improve motor function in patients with PD significantly, and regular physical activity and exercise may potentially slow the progression of disease [3–5]. More recently, novel modifications of conventional physical therapy—such as dance therapy—have demonstrated improved motor outcomes for persons with PD [6], as supported by randomized trials of community-based dance therapy [7–9].

Therapeutic instrumental music performance may optimize gross movements and fine motor skills of the upper limbs via rhythmic entrainment, similarly to how dance therapy may improve gross lower limb movements, posture, and balance [10–13]. The musical instrument, as a target of motor function, provides auditory, visual, and kinesthetic feedback for behavioral and functional modification [12, 13]. Multiple small clinical trials support the use of music and rhythm-based interventions to address motor, cognitive, and neuropsychiatric aspects of PD [13–22]. These interventions may also decrease the amplitude of hyperkinetic involuntary movements seen as a complication in PD [23]. Additionally, music-based treatment may improve nonmotor aspects of the disease including quality of life measures, emotional rehabilitation, and executive symptoms [15, 17, 18]. Use of voice as a musical instrument in a choir or ensemble may also improve patient-specific deficits, such as impaired voice volume [22, 23]. For example, choral group singing requires one to carefully modulate voice volume, maintain a steady rhythm, and sustain an erect posture with group movements, which can facilitate weight-shifting and trunk rotation [22].

Most persons with PD experience at least some speech impairment during the course of their disease, including hypophonia and dysarthria; this can significantly limit communication and adversely affect self-reported quality of life [24, 25]. Decreased range of lip and tongue muscle motion, reduced respiratory capacity and movement coordination, and decreased jaw movement size and peak velocity have been observed, resulting in decreased voice volume, loss of vocal clarity, and reduced intelligibility [26–28]. A hallmark feature of vocal dysfunction seen in persons with PD is a propensity for the voice to diminish in volume and trail off over time [27]. Extrapyramidal and sensorimotor dysfunctions in PD are thought to result in impaired sensorimotor processing, self-perceived vocal volume, vocal vigilance, and internal cueing, all of which play an important role in PD-related voice deficits [29]. Several of these abnormalities can benefit from Lee Silverman Voice Treatment (LSVT® LOUD [25]), an intensive voice training program that involves 4 hour-long sessions per week for 4 weeks. LSVT has been shown to improve voice volume in multiple studies[25, 30, 31]. In addition to improving speech articulation, intonation, facial expression, and swallowing, LSVT is thought to retrain sensory perception and internal cueing, as well as drive activity-dependent neural plasticity [25, 31, 32]. However, the intensity and frequency of LSVT sessions present a barrier to participation for many PD patients, particularly those who must travel long distances for an LSVT-trained speech and language pathologist. Additionally, the program may not be engaging enough to sustain at-home practice for everyone, which is an essential component of the program conducive to prolonged improvement of speech and voice. Music-based approaches to voice therapy may be able to better sustain PD patient engagement while providing additional benefit for voice as well as quality of life [22]. Singing encourages louder voice production than regular speech, and different song tempos and vocal ranges may improve speech rates, intonation, and timing [33]. Additionally, as singing may involve greater articulation than speaking, it may improve the coordination of respiratory, phonation, and articulation aspects of verbal communication [33, 34].

Several groups have investigated the effect of singing in PD. Haneishi et al. found that a music therapy protocol for voice did not significantly improve speech intelligibility or vocal intensity in 4 PD participants given 3 one-hour (15 minutes of singing) sessions per week for 4-5 weeks (mixed singing and nonsinging phonation exercises) [33]. Di Benedetto et al. studied a group of 20 PD participants who underwent 2 one-hour collective sessions of speech therapy plus 1 two-hour collective choral singing sessions per week [35]. Over the 13-week course of the study, improvement in respiratory function, phonation time, and prosody was noted [35], but vocal loudness was not examined. Evans et al. provided group singing instruction and evaluated 17 people with PD at baseline and every 6 months over up to 2 years (10 were followed for the full 2-year duration and had a complete data set for analysis) in a pilot study [36]. They found improvements in the laryngeal elements of the Frenchay Dysarthria Score over two years; PDQ-39 changes were not statistically significant [36]. Tanner et al. conducted an exploratory study in 28 participants with PD to assess whether a combined vocal pedagogy and voice therapy approach that emphasized vocal effort and included singing as 50% of each treatment session can improve vocal ability after 12 treatment sessions (two 6-week sessions; each participant attended 2 90-minute sessions per week, one in a small group of 7 and another in a larger group of 14) [37]. Home practice was tracked and compliance was variable. Two statistically significant changes in vocal skills (in average *f*0 in the reading task and in maximum intensity range), as well as four clinically significant changes (in average frequency in reading, maximum intensity range, frequency range, and fundamental frequency variation in reading) were found in this study [37]. The authors noted lack of a control group and inability to establish a causal effect between the intervention and the change in measured outcomes as key limitations [37]. Barnish et al. performed a systematic review of 7 studies addressing singing in PD and concluded that singing may benefit the speech in PD, despite some conflicting evidence [38]. They recommended further research to assess wider benefits including on functional communication, cognitive status, motor function, and quality of life. Stegemӧller et al. assigned 27 participants to twice- or once-weekly singing groups and analyzed voice, respiratory, and QoL measures at baseline and after an 8-week intervention [39]. They demonstrated objective improvements in inspiratory and expiratory pressure and phonation duration, though vocal measures investigated (intensity and range) did not improve significantly [39]. Shih et al. assessed the effects of 12 weekly 90-minute group singing sessions, led by a voice and speech therapist/singing instructor on 13 participants via an open-label pilot study [40]. Voice loudness as measured by sound pressure level (SPL, in decibels) at 50 cm during speech did not significantly improve at 1 week or 13 weeks from baseline; secondary outcome measures, including the patient-reported Voice Handicap Index and Voice-Related Quality of Life (VRQOL), also did not improve from baseline [40]. Elefant et al. conducted an open-label repeated measures study to evaluate the influence of a group voice and singing intervention (weekly 60-minute sessions for 20 weeks) on speech, singing, and depressive symptoms in 10 participants with PD [41]. Significant changes were observed for five of the six singing quality outcomes at 10 weeks and 20 weeks, as well as voice range and the Voice Handicap Index physical subscale at 20 weeks [41]. No significant changes were found for speaking quality or depressive symptom outcomes; however, there was an absence of decline on speaking quality outcomes over the intervention period [41]. In a controlled 3-month trial, Tamplin et al. examined the effects of an interdisciplinary singing intervention (ParkinSong) on voice and communication in 75 individuals with mild-to-moderate PD [42]. Weekly and monthly interventions were compared. They demonstrated significant improvements in vocal intensity, maximum expiratory pressure, and voice-related quality of life in comparison to controls [42]. Weekly ParkinSong participants increased vocal intensity more than monthly participants, while this measure declined in nontreatment control groups [42]. No statistical differences between groups on maximum phonation length or maximum inspiratory pressure were observed at 3 months [42]. The study team followed up these 75 participants as well as 44 of their care partners at 12 months and compared weekly ParkinSong, monthly ParkinSong, weekly control, or monthly control groups [43]. They found significant improvements in the primary outcome of vocal loudness (*p*=0.032), with weekly singers 5.13 dB louder (*p*=0.044) and monthly singers 5.69 dB louder (*p*=0.015) than monthly controls at 12 months [43]. ParkinSong participants also showed greater improvements in voice-related QoL and anxiety than controls [43]. Caregivers who attended ParkinSong showed greater reductions in depression and stress scores [43].

In the cross-over study of singing in PD reported here, weekly 1.5-hour group singing classes were conducted over 12 weeks in parallel with a weekly PD support group, which served as a social control. Primary outcome measures included patient-centered and objective measures regarding speech and voice. We hypothesized that structured vocal therapy is (1) sustainable for persons with PD who have varying disease severity in an outpatient setting; (2) will improve self-reported quality of life, self-efficacy, and mood; and (3) will improve objective voice and speech function.

## 2. Materials and Methods

### 2.1. Recruitment

Participants were recruited from multiple regional centers in Maryland including university-based movement disorders' clinics and private practices via flyers. Fifty-seven persons were screened for eligibility criteria. Participants met criteria for Idiopathic Parkinson Disease by UK Brain Bank criteria [1] and did not have dementia based on Montreal Cognitive Assessment (MoCA) screening score of >24 [44]. Exclusion criteria included an inability or unwillingness to participate in structured vocal ensemble groups or decompensated psychiatric disorder (active disabling hallucinations, agitation, and suicidal ideation). All participants completed an informed consent form approved by the Johns Hopkins University School of Medicine Institutional Review Board (IRB00065196, approved 12/22/2015). The trial was registered on ClinicalTrials.Gov (NCT02753621).

### 2.2. Design

**Figure 1:**
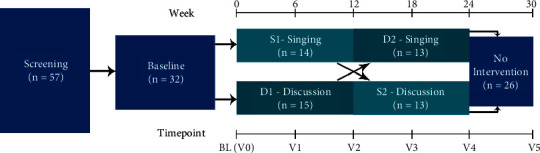
CONSORT diagram of study. Initially, 57 participants were screened for eligibility, resulting in 32 participants randomized to participate. Participants were randomized to two arms: singing-first followed by the discussion group or vice versa. Cross-over from singing to discussion occurred after 12 weeks to increase statistical power. BL, baseline.

This was a prospective randomized, single (assessor)-masked cross-over effectiveness study over 30 weeks. Participants were randomly divided (block randomization via Excel random number generator) into two groups—singing (intervention) versus facilitated discussion group (active comparator)—with weekly meetings over 12 weeks, followed by crossover for an additional 12 weeks (Figure 1). We aimed to improve power to detect group differences by using a crossover design. All participants remained on a stable medication regimen for the duration of the study, and this was monitored at every 6-week outcome assessment visit. A professional choral director led the weekly choir group for 90 minutes per session and taught solfege using Curwen's hand signs [45] and drawing from the Kodály method [46]. Each choir session began with a 10-minute warm-up designed to engage the full body (stretching, sit to stand, and trunk rotation). Rhythmic learning employed pedagogy of Émile Jaques-Dalcroze, Edwin Gordon, Zoltán Kodaály, and Carl Orff. Estill voice training principles were used to develop vocal skills [47, 48]; training included thirteen vocal exercises or “Figures for Voice”: True Vocal Folds: onset/offset control; false vocal folds control; true vocal folds: body-cover control; thyroid cartilage control; cricoid cartilage control; larynx control; velum control; tongue control; aryepiglottic sphincter control; jaw control; lips control; head and neck control; and torso control [47]. Singing groups included home-based vocal training exercises to reinforce the weekly choir sessions. Singing repertoire included Auld Lang Syne (Traditional), Rock My Soul (Peter, Paul, and Mary), I'll Never Fall in Love Again (Burt Bacharach), Raindrops Keep Fallin' on My Head (Burt Bacharach), Edelweiss (Rodgers and Hammerstein), Do-Re-Mi (Rodgers and Hammerstein), Oh, What a Beautiful Mornin' (Rodgers and Hammerstein), You'll Never Walk Alone (Rodgers and Hammerstein), Feelin' Groovy (Simon and Garfunkel), Oh What a Beautiful Morning, and Yellow Submarine (The Beatles). The weekly support group was led by a licensed social worker with PD support group experience who engaged in semistructured discussion with supplementing weekly reading material for 90 minutes per session. Participants were encouraged to discuss any issues of concern to them. Several structures were used to stimulate discussion. Each participant was given two books, both by Dr. Michael S. Okun, *Parkinson's Treatment: 10 Secrets to a Happier Life* and *Breakthrough Therapies for Parkinson's Disease*. Special attention was paid to Chapter 10 of the first book entitled *Kindle Hope into Happiness and a Meaningful Life*. In both groups, participants were asked to bring an object of importance and to share with the group about what the object meant in their lives. Two videos, “Alive Inside” and “Capturing Grace,” were used to stimulate discussion. Both films focus on the role of music and dance in addressing neurological illness. Finally, several specific issues that were identified by the group as areas of concern were addressed. Those issues centered on travelling with disabilities and the balance between marital partners in the care partnering relationship. Due to space considerations, additional details of the singing and discussion group intervention are available per request from the corresponding author.

Both the choir and the control group interventions took place in person concurrently in separate rooms (auditoria) in a single community-based church space (the participants from the two groups did not interact with each other during sessions). Participants who took part in singing intervention first were designated as group “S” (with the 12-week period of singing defined as S1 and the 12-week period of discussion group participation defined as S2) and those who took part in the singing intervention after attending the discussion group were designated as group “D” (with the 12-week period of discussion group participation defined as D1 and the 12-week period of singing defined as D2) (Figure 1).

### 2.3. Outcomes

Primary outcomes assessed were attrition rate (we aimed for at least 80% of participants completing the 30-week study) and objective measures of vocal function. These included volume/loudness (decibels), held vowel duration, jitter, shimmer, and harmonic-to-noise ratio. Of note, minimum and average vocal volume are plausibly linked to conversational voice volume, with multiple aspects of the minimal loudness measure rendering it especially clinically meaningful. Self-reported primary outcomes include the Parkinson's Disease Questionnaire 39 [49] and Voice-Related Quality of Life (VRQOL) scores [51]. Secondary outcome measures included MDS-UPDRS [50], Montreal Cognitive Assessment (MoCA) [44], Geriatric Depression Scale (GDS) [51], Lorig self-efficacy scale [52], and the Short Form-36 (SF-36) [53]. Participants were assessed at baseline and at 6-week increments until the concluding visit at 30 weeks after baseline. MDS-UPDRS Part III (Motor) ratings were performed by an assessor (AP) who was unaware of the participants' group (S or D) assignment. Participants were instructed to aim for their best ON state and were assessed within 60–90 minutes of their last dopaminergic medication dose. Study assessments were carried out between the late morning and early afternoon.

### 2.4. Vocal Data Acquisition

Each participant was seated at a table with a recording device (R-05 Wave/MP3 recorder, Roland Corporation, Shizuoka, Japan) and portable electronic sound pressure level meter (SPER Scientific model 850013; Scottsdale, AZ, USA) that were both placed 50 cm from the participant's mouth. Audio recordings were obtained during 1 : 1 assessment sessions behind closed doors, in a church room separate from the auditoria where the study interventions took place, thereby minimizing noise. Sound pressure level readings and audio recordings were acquired at 6 timepoints, every 6 weeks. SPL readings were stored as. rec files and audio recordings were stored as waveform audio (.wav) files on a password-protected online server accessible only to the study team. For testing, participants were asked to (1) read the standardized “Rainbow” passage, (2) describe the standardized “Cookie Theft” picture at an everyday speaking volume for 30 seconds, and (3) enunciate and hold the vowel sounds “A” and “E” for as long as possible on one breath; 3 trials for each vowel sound assessed maximal volume, and 3 additional trials for each sound assessed everyday speaking volume. Averages across the 3 trials were used for analysis.

### 2.5. Vocal Analysis

Volume range (minimum, maximum, and average) for the sound pressure level readings was read directly from the SPL meter for the above voice measures.

The .wav file audio recordings were analyzed for held vowel, Rainbow passage, and Cookie Theft picture description duration. They were also analyzed using Praat software (Paul Boersma and David Weenink, Praat: doing phonetics by computer. Version 5.4.01 from https://www.praat.org/) for formant analysis (measure of voice resonance) and spectral analysis. The following quantitative sonographic metrics were derived: voice jitter, shimmer, and harmonic-to-noise ratio (HNR).

### 2.6. Statistical Analysis

**Figure 2:**
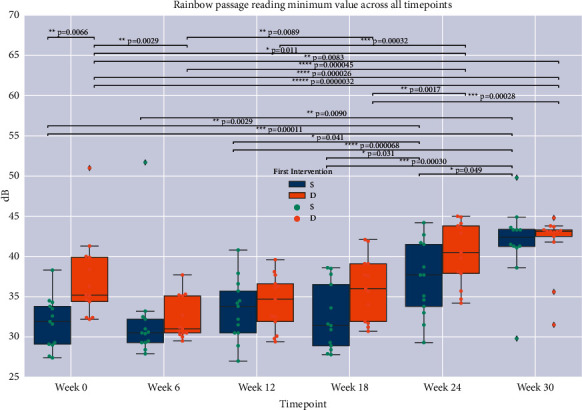
Boxplots of Rainbow S (singing first) and D (discussion) groups' minimal loudness (in decibels, dB) for all timepoints. Boxes show medians and interquartile range (IQR) and whiskers show largest data value within 1.5 IQR above the third quartile and smallest data value within 1.5 IQR below the first quartile. Brackets and asterisked *p* values indicate statistical significance.

**Figure 3:**
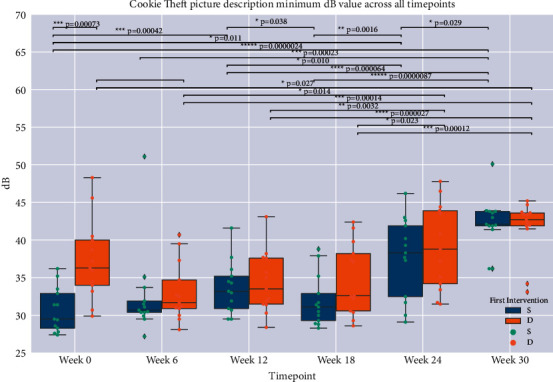
Boxplots of Cookie Theft S (singing-first) and D (discussion-first) groups' minimal loudness, all timepoints. Brackets and asterisked *p* values indicate statistical significance.

**Figure 4:**
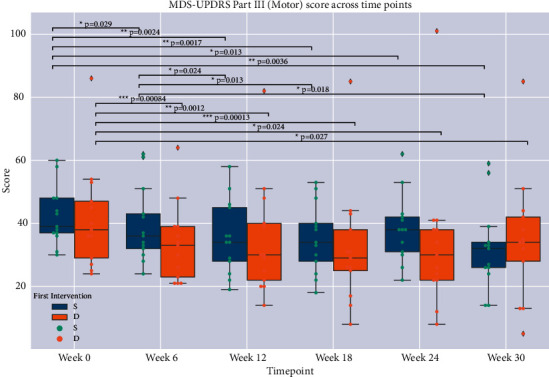
Boxplots of MDS-Unified Parkinson Disease Rating Scale Motor (UPDRS III) S (singing first) and D (discussion first) group scores. Brackets and asterisked *p* values indicate statistical significance.

Descriptive statistics included means and standard deviations for all outcome measures. Comparison of mean baseline values to those at each successive timepoint was performed on the entire cohort and within groups S (singing first) and D (discussion group first). Mann–Whitney *U* and Fisher's exact tests were used to compare differences between groups based on order of intervention (Table 1). Mixed linear models were employed to examine the effects of (a) intervention, (b) intervention order adjusted for time from baseline, and (c) intervention and order adjusted for time from baseline (Tables 2–5). Outcome measures that passed screening with ANOVA *F*-test *p* values below the threshold for Hochberg False Discovery Rate (FDR, correction for multiple comparisons = 0.05) [54] were further assessed by post-hoc, two-sample *t*-tests (Figures 2–4). Pearson's correlation coefficients were derived for linear regression performed on vocal measures on clinical and functional outcome scores. Statistical significance was set at *p* < 0.05. We attempted to account for baseline differences by (1) using within-group analysis of score changes over time (for groups S and D separately), as presented in Figures 2–4, and by (2) using intervention order adjusted for time from baseline as a covariate in our mixed linear models, as presented in Tables 2–5.

## 3. Results

### 3.1. Recruitment

Fifty-seven participants were screened, and 32 participants from the Greater Baltimore area were enrolled based on eligibility criteria. Twenty-six participants (81%) completed the study. Three participants dropped out prior to the first session, two prior to intervention cross-over, and one before study conclusion. Reasons stated for dropping out included transportation difficulty, reduced ambulatory ability, and intercurrent medical illness. Twenty of 26 final participants expressed the desire to continue with the singing program and opted to continue singing weekly under the same choir leadership after study conclusion (see Discussion). Despite block randomization, there were significant baseline differences between groups in age, gender distribution, disease severity (Hoehn & Yahr stage), and several volume measures (Table 1). Group S had a slightly less advanced disease stage, a lower proportion of women (4F : 9 M in group S vs. 6F : 7 M in group D), and decreased loudness across multiple voice measures at baseline (Table 1). There were no baseline MDS-UPDRS motor score differences between the groups (Group S week 0 score 42.2; Group D week 0 score 41.5, *p* > 0.05).

### 3.2. Intention-to-Treat Analyses

#### 3.2.1. Speaking Volume, Motor Function, and Other Outcomes in Overall Cohort

**Figure 5:**
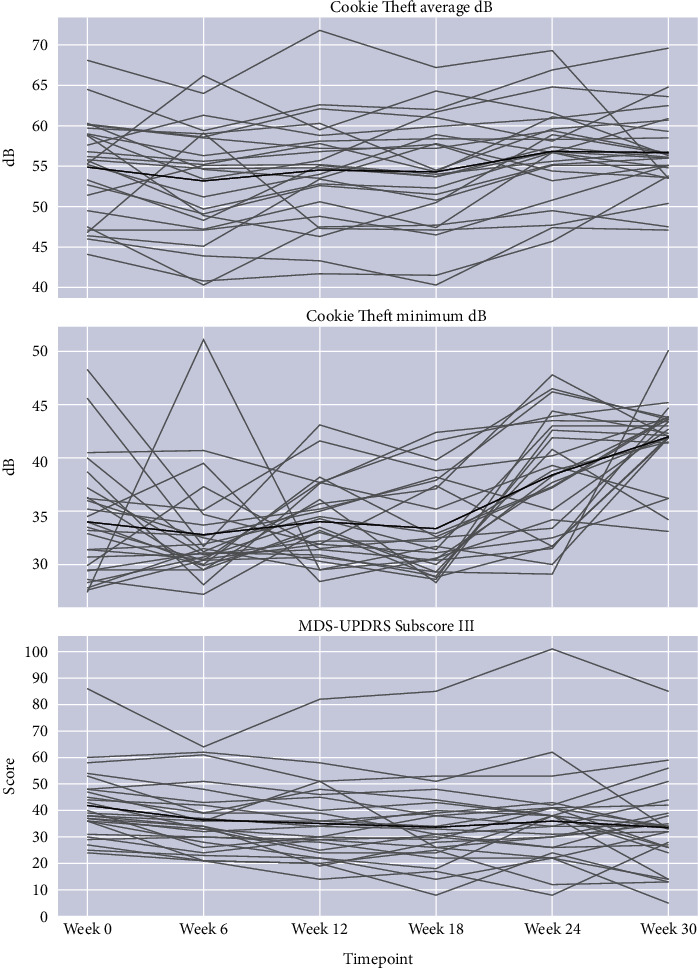
Spaghetti plots showing (a) Cookie Theft average loudness (in decibels, dB) at each timepoint, (b) Cookie Theft minimal loudness at each timepoint, and (c) MDS-Unified Parkinson Disease Rating Scale Part III (motor) scores at each timepoint for the entire study cohort (*n* = 26).

*t*-test analyses of speaking volume showed significant improvement while describing the Cookie Theft picture for (a) average volume at 24 weeks (mean delta or Δ = 2.06 dB, *p* = 0.004), (b) minimal volume at 24 (Δ = 4.4 dB, *p* = 0.001) and 30 weeks (Δ = 8.1 dB, *p* = 0.001). MDS-UPDRS Part III (motor section) score showed significant improvement at 24 weeks (Δ = 5.9 points, *p* = 0.002) and 30 weeks (Δ = 8.4 points, *p* = 0.001) in the overall cohort. In the mixed linear model analyses, when the baseline was adjusted to week 12 for group D (to parallel the immediate presinging baseline for group S at week 0), the changes in the overall cohort at these time points became nonsignificant (see Table 2 for voice and Table 4 for MDS-UPDRS outcomes; see Figure 5 for individual data at all timepoints for average and minimal loudness while describing the Cookie Theft picture and MDS-UPDRS total scores).

Mixed linear model analysis demonstrated improvements in the Emotional Well-Being (mean delta −12.7 points, *p*=0.037) and Body Discomfort (mean delta −18.6 points, *p*=0.001) domains of the PDQ-39 from baseline to week 24 in the overall cohort. There were additional improvements observed from baseline to week 12 for the Stigma domain (mean delta −12.9, *p*=0.046).

There were no significant changes observed in the overall cohort for other voice measures, VRQOL, SF-36, GDS, or MoCA scores.

Analyses were performed to compare different effects between the orders of intervention as a function of time from the start of intervention. The changes in measured values over the course of the two interventions in each group were also compared.

### 3.3. Per-Protocol (Group S and Group D) Analyses

#### 3.3.1. Changes after 12-Week Singing Intervention and beyond (Figures 2–4, *t*-Test Analyses)

*t*-test analyses demonstrated that group S (singing first) saw significant improvement in Cookie Theft picture description minimal volumes (mean Δ = 2.74 dB, *p* < 0.001) during the 12 weeks of singing intervention, and a significant sustained improvement from baseline in the same measure at 24 weeks (Δ = 6.96 dB, *p* < 0.001) and 30 weeks (Δ = 11.59 dB, *p* < 0.001). Group D (discussion first) also saw significant improvement during the 12 weeks of singing intervention in both reading (Rainbow passage) and picture description (Cookie Theft) minimal volume (resp., Δ = 6.14 dB, *p* < 0.001, Δ = 5.18 dB, *p*=0.0032), lasting to the end of the trial, 18 weeks after beginning of singing intervention (resp., Δ = 7.47 dB, *p* < 0.001, Δ = 7.32 dB, *p* < 0.001).

Group S (singing first) saw improvement in average total MDS-UPDRS III (motor) scores during the 12 weeks of singing intervention (Δ = −6.69, *p*=0.0024). This change persisted to the conclusion of the trial at 30 weeks (Δ = −10.15, *p*=0.0036). Group D (discussion first) saw improvement during the 12 weeks of discussion intervention (Δ = −6.92, *p*=0.0012) as well as from baseline to the final visit of the trial at 30 weeks (Δ = −6.62, *p*=0.027). The D group did not see statistically significant improvement over the course of their singing intervention, nor did the S group over the course of their discussion intervention.

#### 3.3.2. Changes during Singing Intervention versus the Discussion Group (Tables 2–5, Mixed Linear Model Analyses)

Comparative assessment of the changes (Δ) in vocal measures observed during singing versus during discussion interventions was performed using mixed linear modeling. Group D (singing second) had greater improvement from presinging baseline (week 12) to week 24 (completion of intervention) than group S for Cookie Theft average loudness (mean difference in Δ between groups 3.2 dB, *p*=0.037) and Rainbow passage minimum loudness (mean difference in Δ between groups 6.5 dB, *p*=0.001). For MDS-UPDRS, there was a trend toward greater improvement on Part II (Motor Aspects of Experiences of Daily Living) for group S versus group D from start to end of interventions (−3.6 points, *p*=0.051) but no other significant differences. For PDQ-39, there were significantly greater improvements for group S versus group *D* for the communication (mean difference in Δ between groups −12.9, *p*=0.017) and body discomfort (mean difference in Δ between groups −19.8, *p*=0.001) domains from beginning to end of interventions (week 24).

There were no other significant improvements in sustained vowel duration, derived quantitative sonic measures (HNR, Jitter, and Shimmer), cognition, mood, self-efficacy, voice-related quality of life, or health-related quality of life observed over the course of the study.

#### 3.3.3. Age and Hoehn & Yahr Stage Correlate with Vocal Function

Hoehn & Yahr stage correlated weakly with the change over the course of S and D groups' singing and intervention periods (*R*^2^ was <0.26 for all voice measures, *p* > 0.05). Age showed a somewhat stronger correlation (*R*^2^ > 0.5) for Rainbow Paragraph reading duration and Cookie Theft description HNR, but this was nonsignificant. When considered together with disease stage, the parameters displayed a strong correlation with vocal dysfunction, especially for the Cookie Theft Shimmer measure (*R*^2^ = 0.80, *p* < 0.001).

## 4. Discussion

In this study, group singing was evaluated as a therapeutic intervention for speaking, motor function, and quality of life in PD. Overall, participants showed significant improvements in conversational speaking volumes (minimum and average dB). Notably, minimal volume for both passage reading and conversational picture description increased significantly during singing intervention in the discussion-first group D. This improvement was also observed in the singing first group S, which continued to improve after cross-over to discussion group. The degree of improvement we observed is comparable to that observed for the well-established PD-specific speech therapy modality LSVT LOUD®, where average volume increases by about 8 dB from baseline after 4 weeks (end of intervention) and is sustained at +6 dB at 6 months [25, 31]. Due to the propensity of patients with PD to trail off in volume during everyday speech and their ability to generate maximal loudness on demand [27], the minimum loudness improvements observed in this study for both the Cookie Theft picture description and the Rainbow Passage reading (Table 1 and Figures 2 and 3) are clinically relevant. This is especially true for group S, which was significantly more impaired on these voice measures than group D at presinging baseline (i.e., may have had more to gain from both study interventions) and achieved similar minimal loudness levels as group D at weeks 24 and 30 (Table 1). Group S was at a less affected Hoehn & Yahr stage. It is also possible that there was a ceiling effect on minimum voice loudness from the interventions studied here, which may explain why group S and group D reached very similar loudness measures at week 30 (Table 1); longer-term studies are required to clarify this.

We also observed significant improvements from baseline to week 24 (intervention completion) in PDQ-39 Body Discomfort and Emotional Well-Being domains for the overall cohort. The magnitude of these improvements was well above the minimal clinically important difference of about 5 points per domain for PDQ-39 suggested in prior literature [24, 49, 55]. While Communication (mean delta −7.5 points) and Cognitive Impairment (mean delta −5.0 points) domains of PDQ-39 also had clinically meaningful improvements from baseline to week 24 in the overall cohort, they did not reach statistical significance; in addition, these changes were not accompanied by statistically significant improvements in voice-related quality of life, as consistent with Shih et al. [40] and in contrast to the controlled ParkinSong study [42]. Thus, larger studies are needed to clarify whether group singing can improve self-reported impairments in cognitive function and communication in PD.

Comparative analysis between groups in our study had significant limitations, as participants in both groups S and D may have been self-motivated to continue singing practice on their own after conclusion of the singing intervention (despite explicit instruction not to do this between completion of interventions at week 24 and the final assessment at week 30 for the entire cohort, weeks 12–30 for group S, and 0–12 and 24–30 for group D). This may explain the continued improvement seen later in the singing-first group during their discussion group period and the additional improvements observed on several outcome measures in our study from week 24 to week 30. We thus note that it is crucial to carefully monitor ongoing singing practice/choir participation after completion of study interventions. The discussion groups had a support group format and intentionally had an active social component, in addition to encouraging voice projection among group members. The discussion group period was intended to attenuate prosocial confounding present in any group interventions in PD but did not fully eliminate observational bias such as the Hawthorne effect (the participants' awareness of being observed affecting their performance) and the effects of expectation. Therefore, we cannot conclusively attribute the significantly greater effect size (Cohen's D effect size metric, EF) of singing versus discussion intervention (Rainbow min dB EF = 1.89, Rainbow avg dB EF = 1.57, Cookie min dB EF = 0.26, and Cookie avg dB = 0.70) in the discussion-first group specifically to the singing intervention's effectiveness in improving conversational volume in PD. Similarly, we cannot conclusively attribute MDS-UPDRS motor improvements to the singing intervention (this was not an expected outcome of the study), as UPDRS motor scores are subject to significant natural visit-to-visit variability in PD.

Additional limitations of our study included a relatively small sample size and block randomization that resulted in significant between-group differences in baseline disease severity and voice function. Randomization stratified by age, gender, and disease duration should be considered for future studies. We did not have complete data on disease duration for our cohort, which is a limitation because differences in disease duration could have explained some of the baseline differences between groups S and D. Additionally, while the study followed all participants for an equal time duration, due to the cross-over design, follow-up after cessation of singing intervention was shorter in the discussion first group. While the most marked functional improvements were seen at 18–24 weeks following intervention, this was only seen in the singing-first group. The same improvement, however, was observed in the discussion-first group at an earlier timepoint. Due to these complicating factors for analysis, we advise against using crossover design in future studies of therapeutic singing interventions in PD, instead focusing on comparing interventions delivered in parallel. We recognize the need for future multicenter studies with more participants assessed over a period of at least 52 weeks (24 weeks after intervention); we also note the need to evaluate other clinically meaningful outcome measures in addition to quantitative vocal and respiratory measurements, including swallow function. This was previously done by Stegemöller et al. [34], who found a significant increase in surface EMG swallow-related outcome measures, as well as significant improvements in UPDRS total and UPDRS motor scores in an open-label 8-week study of group singing in 24 participants with PD (18 participants attended weekly singing sessions while 6 attended twice per week; no effects of increased singing frequency were found) [34]. Also, no significant differences were revealed for SWAL-QOL in this study [34]. Importantly, we did not directly assess social functioning and functional communication in our study, which limits real-world application of these types of interventions [38]. In this regard, multimodal interventions that include voice training, such as active theater therapy, have shown promise toward improving social functioning and emotional well-being in PD [56, 57]. Finally, practice effects and increased familiarity with the outcome assessments performed every 6 weeks may have contributed to improvements over time as well.

The singing-first group had a slower initial improvement in speaking volume from the singing intervention, taking up to 18–24 weeks. While it may be that singing interventions take at least 18 weeks to show significant improvement, our study was not able to answer this question definitively because there was unequal postsinging follow-up time between groups (due to the crossover design) and some of the observed improvements could have resulted from the Discussion group and from increased familiarity with the outcome assessments.

The observed increase in the minimal loudness of reading and picture description suggests that the participants' voice loudness at the quietest point of conversational speech may increase as well, lessening the degree to which participants' voices taper off from sustained speech. This is consistent with the observed improvements in average loudness for the held “A” vowel sound. These results suggest that the overall cohort of participants experienced a meaningful improvement in conversational voice volume from baseline to the end of the study.

The improvement in respiratory function shown by several prior studies may underlie the improvement our data show in the clinically meaningful vocal loudness measures [39, 42]. However, as we show significant improvement only 18–24 weeks after intervention, the duration of most prior studies may have been too short to yield similar improvements in vocal volume. Nevertheless, previous studies demonstrated improvement in certain quantitative measurements, and the clinically meaningful improvements we were able to observe over the course of our 30-week study expand upon and add to prior work [33, 35–43].

Comparison of singing and discussion interventions showed that some voice improvements were specifically attributable to the patients' participation in the group singing interventions. This group singing-driven improvement in objective measures central to conversational voice volume was clinically meaningful for PD patients. This benefit was noticeable to the participants themselves, as most (20/26 participants, or 77%) requested to continue choir participation after the completion of the study. The ParkinSonics choir continues to meet weekly throughout the year (currently via Zoom during the COVID-19 pandemic) and has expanded to include patients with other parkinsonian disorders and care partners.

## 5. Conclusions

Weekly group singing therapy may improve minimum and mean speaking volume and some quality-of-life aspects in PD, showing improvements at 24 weeks from the start of intervention. Our study design limited our ability to attribute improvements in outcome measures specifically to the singing intervention. It may take 18–24 weeks to demonstrate measurable improvements after singing interventions, and further research is necessary to confirm the clinically meaningful improvements found in our study. Multicenter randomized controlled studies are needed to compare the benefits of singing, support group attendance, and speech therapy in PD Figure5.

## Figures and Tables

**Table 1:** Demographics, vocal, and VRQOL measures.

Measure	Singing first-S (*n* = 13)	Discussion first-D (*n* = 13)	*P* value
Age (years)	70.5 (6.9)	66.7 (10.1)	**<0.001**
Gender	9 male/4 female	7 male/6 female	**<0.001**
Hoehn & Yahr stage	2.15 (0.36)	2.42 (0.43)	**<0.001**
Cookie theft (CT) average (avg) loudness week (wk) 0 (dB)	51.9 (5.5)	57.9 (5.3)	**0.012**
CT avg loudness wk 12	52.1 (5.6)	57.0 (6.6)	0.057
CT avg loudness wk 24	54.7 (5.6)	59.0 (5.1)	*0.050*
CT avg loudness wk 30	55.7 (4.5)	57.7 (5.4)	0.139
CT minimal (min) loudness wk 0	30.7 (3.0)	37.3 (5.4)	**<0.001**
CT min loudness wk 12	33.7 (3.5)	34.3 (4.0)	0.687
CT min loudness wk 24	37.7 (5.4)	39.1 (5.7)	0.479
CT min loudness wk 30	42.3 (3.5)	41.7 (3.7)	1
CT maximal (max) loudness wk 0	67.4 (5.1)	71.4 (5.0)	0.081
CT max loudness wk 12	66.1 (4.8)	71.5 (4.9)	**0.012**
CT max loudness wk 24	66.7 (5.5)	71.2 (5.8)	0.081
CT max loudness wk 30	67.3 (4.5)	69.9 (5.2)	0.223
Rainbow passage (RP) avg loudness wk 0	55.0 (7.6)	60.8 (4.8)	0.057
RP avg loudness wk 12	53.4 (9.3)	60.8 (5.2)	**0.039**
RP avg loudness wk 24	56.5 (6.5)	61.3 (4.9)	0.057
RP avg loudness wk 30	57.3 (6.0)	61.1 (4.2)	0.123
RP min loudness wk 0	31.8 (3.2)	37.2 (5.2)	**0.001**
RP min loudness wk 12	33.4 (3.8)	34.3 (3.4)	0.511
RP min loudness wk 24	37.0 (4.6)	40.4 (4.0)	*0.050*
RP min loudness wk 30	41.8 (4.7)	41.7 (3.8)	0.852
RP max loudness wk 0	69.5 (5.5)	73.6 (4.3)	0.081
RP max loudness wk 12	69.4 (7.3)	74.1 (5.0)	0.081
RP max loudness wk 24	73.7 (10.6)	73.5 (6.1)	0.687
RP max loudness wk 30	71.2 (8.6)	73.6 (5.6)	0.32
Voice-related quality of life (VRQOL) total wk 0	18.8 (8.3)	21.7 (9.5)	0.479
VRQOL total wk 12	19.4 (7.4)	23.7 (10.4)	0.264
VRQOL total wk 24	20.8 (10.5)	22.2 (7.9)	0.538
VRQOL total wk 30	16.6 (5.4)	21.75 (10.5)	0.266

**Table 2:** Voice outcomes.

Comparison (adjusted for presinging baseline)		Cookie Theft picture average loudness in decibels (dB)			Cookie Theft picture minimum loudness (dB)			Cookie Theft picture maximum loudness (dB)			Cookie Theft picture loudness range (dB)			Rainbow passage average loudness (dB)			Rainbow passage minimum loudness (dB)			Rainbow passage maximum loudness (dB)			Rainbow passage loudness range (dB)		
		dB Δ	*P* value	*R* ^2^	dB Δ	*P* value	*R* ^2^	dB Δ	*P* value	*R* ^2^	dB Δ	*P* value	*R* ^2^	dB Δ	*P* value	*R* ^2^	dB Δ	*P* value	*R* ^2^	dB Δ	*P* value	*R* ^2^	dB Δ	*P* value	*R* ^2^
Overall cohort (weeks compared)	12–BL (week 0)	0.48	0.732	0.046	2.38	0.166	0.598	−2.76	0.051	0.208	−6.40	<0.001	0.550	−1.23	0.400	0.050	0.70	0.671	0.561	−0.19	0.880	0.037	−4.47	0.036	0.369
	24–12	−0.87	0.591	0.244	−0.94	0.622	0.032	−0.07	0.976	0.059	1.71	0.472	0.025	0.02	0.983	0.490	−3.28	0.069	0.403	3.21	0.258	0.160	6.64	0.117	0.153
	24–BL	0.53	0.692	0.155	2.03	0.349	0.307	−1.20	0.439	0.050	−5.66	**0.010**	0.242	−0.14	0.904	0.214	−0.80	0.609	0.302	3.79	0.192	0.086	2.17	0.538	0.017
Singing 1st (S) vs. singing 2nd (D)	Δ from start (presinging BL) to end interventions (week 24)	−3.19	0.037	0.282	−2.36	0.208	0.081	−2.03	0.203	0.112	0.67	0.711	0.077	−2.04	0.139	0.091	−6.05	**0.001**	0.416	0.82	0.586	0.021	4.50	0.083	0.196
	Δ final visit (week 30) vs. end interventions (week 24)	1.93	0.248	0.432	0.79	0.652	0.705	0.36	0.794	0.328	−3.40	0.088	0.330	1.12	0.284	0.709	2.92	0.176	55.610	1.46	0.458	0.104	−6.42	0.080	0.250

**Table 3:** VRQOL total scores.

Comparison		Δ	*P* value	*R* ^2^
Overall cohort (weeks compared)	12–BL	−1.9	0.423	0.096
	24–12	0.9	0.738	0.256
	24–BL	−0.1	0.984	0.284
Singing 1st vs. singing 2nd	Δ from start to end intervention	0.4	0.839	0.355
	Δ final visit vs. end intervention	−3.6	0.099	0.124

**Table 4:** MDS-UPDRS subscales.

Comparison (adjusted for presinging baseline)		Part 1			Part 2			Part 3			Part 4		
		Δ	*P* value	*R* ^2^	Δ	*P* value	*R* ^2^	Δ	*P* value	*R* ^2^	Δ	*P* value	*R* ^2^
Overall cohort (weeks compared)	12–BL	−0.4	0.839	0.006	−3.6	0.058	0.168	0.7	0.748	0.018	−2.7	0.109	0.358
	24–12	−1.4	0.370	0.354	−0.3	0.888	0.011	3.0	0.435	0.029	−1.4	0.275	0.142
	24–BL	−0.7	0.676	0.065	−3.3	0.118	0.096	3.7	0.285	0.11	−2.4	0.221	0.246
Singing 1st vs. singing 2nd	Δ from start to end interventions	−1.5	0.404	0.281	−3.6	0.051	0.171	−6.9	0.110	0.164	−1.0	0.427	0.264
	Δ final visit (week 30) vs. end interventions (week 24)	−0.7	0.620	0.018	−1.9	0.133	0.105	−3.9	0.350	0.189	−1.2	0.303	0.106

**Table 5:** PDQ-39 Quality of Life Outcomes.

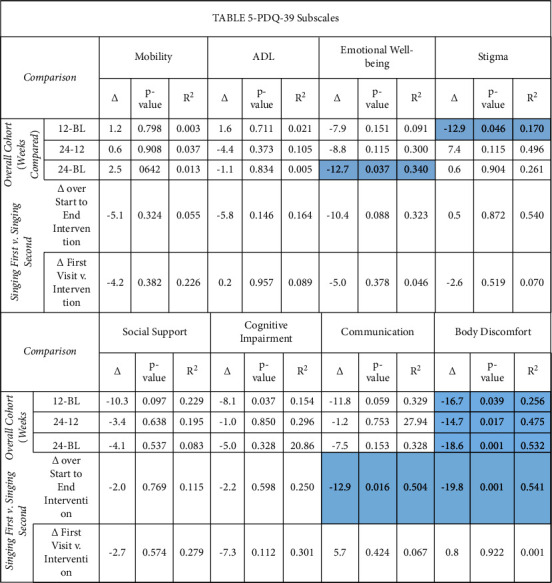

## Data Availability

The data used to support the findings of this study have been restricted by the Johns Hopkins Hospital Institutional Review Board to protect participants' privacy and are available from the corresponding author upon request.

## Supplementary Materials

Supplementary Materials SuppFig1_VocalMeasuresBoxplots_exported.jpg: Boxplots for deltas (changes) from singing and discussion interventions for all vocal outcome measures. SuppFig2_VocalMeasuresSpaghplots_exported.jpg: Spaghetti plots for all vocal outcome measures over the course of the study, further separated into S and D groups.
