# Supplementary material for: Parkinsonics: A Randomized, Blinded, Cross-Over Trial of Group Singing for Motor and Nonmotor Symptoms in Idiopathic Parkinson Disease
Source: Parkinsons Dis. 2022 Sep 20;2022:4233203. doi: 10.1155/2022/4233203 (PMC9553721; doi:10.1155/2022/4233203)

Deltas in outcome measures from each intervention in singing and discussion first groups

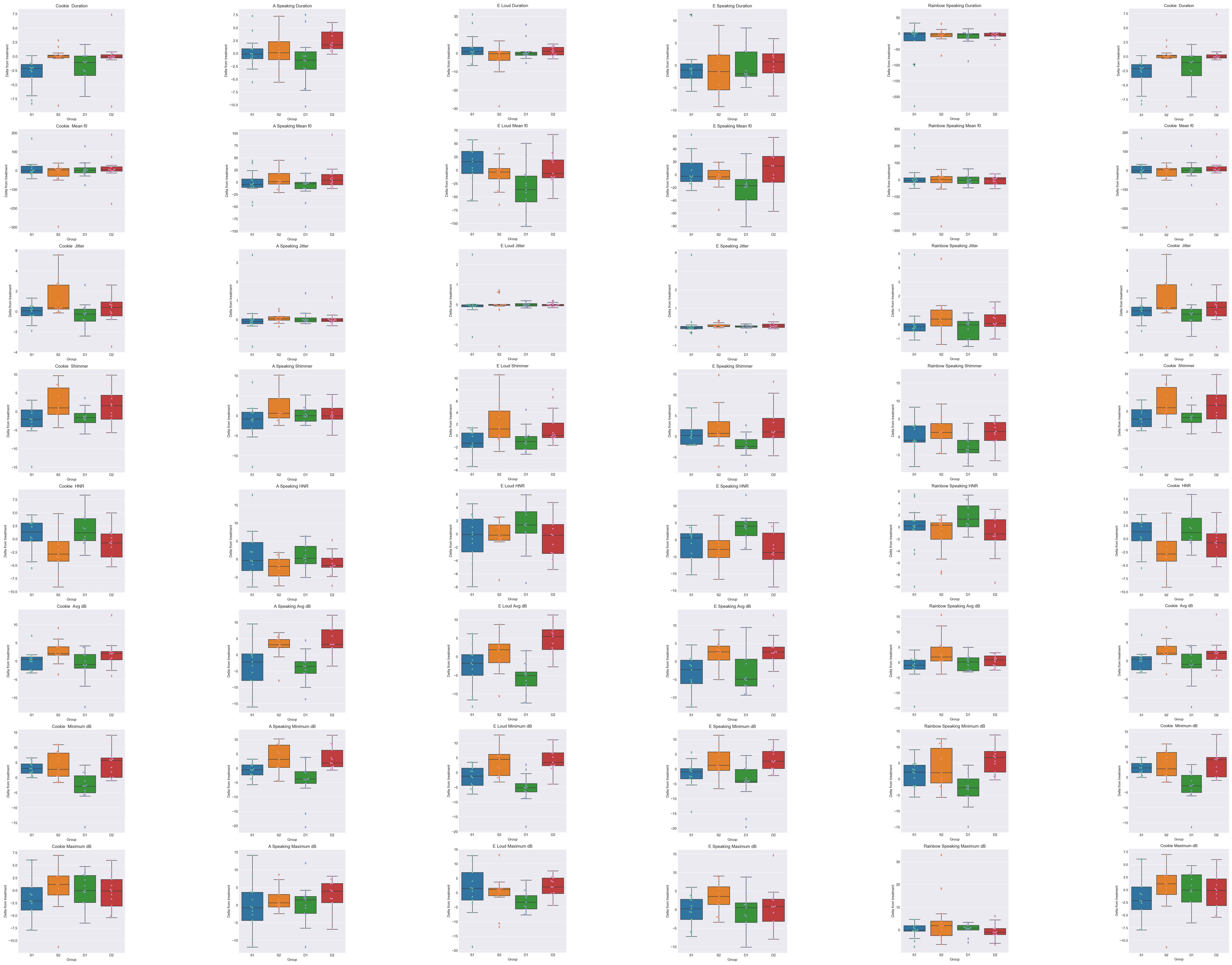

Supplement: Supplementary Materials — SuppFig1_VocalMeasuresBoxplots_exported.jpg: Boxplots for deltas (changes) from singing and discussion interventions for all vocal outcome measures. SuppFig2_VocalMeasuresSpaghplots_exported.jpg: Spaghetti plots for all vocal outcome measures over the course of the study, further separated into S and D groups. [file SuppFig1_VocalMeasuresBoxplots_exported.pdf › 4233203.f1/SuppFig1_VocalMeasuresBoxplots_exported.pdf]
